# Reply to: “Considerations about the use of glucometers for testing glucose tolerance”

**DOI:** 10.1515/almed-2024-0169

**Published:** 2024-12-02

**Authors:** Estéfani Martínez-Chávez, Blanca Fabre-Estremera, Marta Manzano Ocaña, Pilar Fernández-Calle, Antonio Buño Soto, Paloma Oliver

**Affiliations:** Department of Laboratory Medicine, 16268 La Paz University Hospital, Madrid, Spain

**Keywords:** testing glucose tolerance, glucometers, glucose

To the Editor,

We thank Lavin-Gomez B.A. and Guerra Ruíz A.R. [1] for their valuable comments to our paper titled “*Use of point-of-care glucometers during an oral glucose tolerance test in children for prediabetes and diabetes diagnosis: A comparison study*” [2]. We are extremely grateful for your interest in our study and for your suggestions, which substantially contribute to the debate on this issue. Please, find below our response to your considerations.

**Figure 1:**
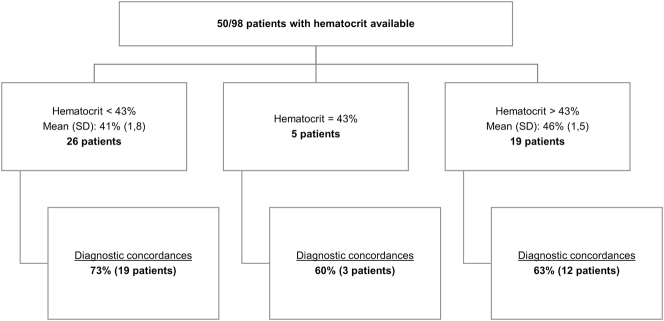
Diagnostic concordance between POCT_ACI_ and the central laboratory as a function of hematocrit values. Distribution of the 50 patients with hematocrit values available. Analysis of diagnostic concordance between POCT_ACI_ and the central laboratory. Hematocrit values were categorized into three groups: hematocrit<43 % (mean 41 %, SD: 1.8, diagnostic concordance 73 %); hematocrit=43 % (diagnostic concordance 60 %) and hematocrit>43 % (mean 46 %, SD: 1.5, with a diagnostic concordance of 63 %).

(1) Pre-analytical sample handling: Thank you for your observation. We would like to clarify that serum tubes with gel are used in our center during the oral glucose tolerance test. A centrifuge is available in the Unit of Diabetes to minimize the effects of *in vitro* glycolysis. In our paper, we acknowledge the potential influence of *in vitro* glycolysis on fasting samples as a limitation. Thanks to the study, our sample handling protocol was optimized to ensure that fasting samples are centrifuged within 20 min of collection.
(2) Correlation between glucose measurements: Comparative analysis of the methods revealed that the differences observed in glucose results obtained with the POCT_ACI_ (Accu-Chek^®^ Inform-II, Roche Diagnostics, Basel, Switzerland) and the central laboratory (Atellica^®^Solution-CH; Siemens Healthineers, Erlangen, Germany) were not statistically significant. These differences complied with our quality specifications for glucose concentrations of 100, 125, 140 and 200 mg/dL. However, in the light of the clinical impact of diagnostic discrepancies, we decided to examine the diagnostic concordance between the two methods.
  The results revealed that differences in glucose concentrations between POCT_ACI_ and the central laboratory tended to increase as glucose concentrations rose. Thus, the POCT glucometer yielded lower results than the central laboratory at higher glucose concentrations. Due to the study design, the reason for lower glucometer results at 30, 60 and 120 min, as compared to laboratory results, could not be determined. Future research that controls for additional variables may help explain these observed differences.
(3) Interference of hematocrit in glucose measurement: The POCT_ACI_ and POCT_ACP_ glucometers (Accu-Chek^®^ Performa, Roche Diagnostics, Basel, Switzerland) used in our laboratory do not measure hematocrit. Given the interest in this variable, we examined hematocrit results in our study population. Of the 98 patients included, a hemogram was included in the laboratory test request of 50 patients. POCT_ACI_ and laboratory-based diagnoses were consistent in 68 % (34) of cases (see Figure 1).
  Separate analysis of diagnostic discrepancies revealed that in 3 of the 4 patients with hematocrit results >43 % (range 44–49 %), diagnostic discrepancies could have had a clinical impact. In all cases, the results obtained with the glucometer were lower than those of the central laboratory. Additionally, in 2 of the 5 cases with hematocrit results <43 % (range 36–42 %), diagnostic discrepancies could have had clinical implications. In the both cases, lower results were obtained with the glucometer, as compared to the laboratory (see Table 1). Therefore, in our study, when hematocrit was <43 %, the inverse relationship between hematocrit and glucose described in the literature [3] was not observed.
  In our study population, we found that glucometer tended to underdiagnose prediabetes and diabetes, except in cases of impaired fasting glycemia. Thus, diagnostic discrepancies in the latter were probably due to *in vitro* glycolysis in fasting samples. In our study, a clear influence of hematocrit was not observed on glucose results.

Next-generation glucometers with capacity to measure hematocrit could yield more precise readings and reduce variations. This would pave the way for the potential use of POCT glucometers for diagnostic purposes in the near future. We once again thank Lavin-Gomez BA and Guerra Ruíz AR for their valuable comments, which contributed to the debate about the use of glucometers in the clinic.

**Table 1:** Diagnostic discordance between POCT_ACI_ and the central laboratory.

POCT_ACI_	Central laboratory	Clinical impact	Hematocrit, %
IFG	Normal	None	36
IFG	Normal		40
IFG	Normal		42
IFG	Normal		43
IFG	Normal		?
IFG	Normal		?
IFG	Normal		?
Normal	IGT		47
IFG	IFG+IGT		?
IGT	Diabetes		?
Normal	IGT	Mild	41
IGT	Diabetes		44
IGT	Diabetes		?
Normal	IGT	Severe	48
Normal	IGT		?
IFG+IGT	Diabetes		47
IGT	Diabetes		41

IFG, impaired fasting glucose; IGT, impaired glucose tolerance; POCT_ACI_, connected glucometer.
